# Advanced uterine adenosarcoma with sarcomatous overgrowth in a young woman: A case report

**DOI:** 10.1097/MD.0000000000018119

**Published:** 2019-11-22

**Authors:** Bin Wang, Hua-Di Yang, Xin-He Shi, Hui Li

**Affiliations:** Department of Gynecology and Obstetrics, The First Affiliated Hospital of Zhejiang Chinese Medical University (Zhejiang Provincial Hospital of Traditional Chinese Medicine), Hangzhou, Zhejiang, China.

**Keywords:** Mullerian adenosarcoma, sarcomatous overgrowth, uterine adenosarcoma, young women

## Abstract

**Rationale:**

Uterine adenosarcoma (UA) with sarcomatous overgrowth (ASSO) is a rare and aggressive disease. Herein, wereported the case of a young patient with advanced uterine ASSO.

**Patients concerns:**

A 29-year-old woman with the diagnoses of endometrial polyp and adenomyosis underwent hysteroscopic endometrial polypectomy for the giant endometrial polyp. Postoperative regular ultrasound scan indicated thickened endometriumand an ill-defined mass with continuous enlargement in the myometrium of the posterior wall of the uterus, which was considered as an adenomyoma. Two years after hysteroscopy, she was re-admitted due to lower abdominal distension and large pelvic mass. At that time, she had taken oral short-acting contraceptives for 2.5 years.

**Diagnoses:**

Magnetic resonance imaging (MRI) of the pelvis revealed an irregular mass with the size of 12∗56∗107 mm^3^ in the right annex area, without distinct border with the rectum, moreover, an uneven intrauterine echo that has no obvious boundary with uterine wall. Right ovarian cancer and adenomyoma were initially considered.

**Interventions:**

The patient received transperitoneal retroperitoneal pelvic combined with total viscera resection, including uterus, bilateral appendages and rectum, omentectomy, appendectomy, lymphadenectomy, and ileostomy. Postoperative pathology confirmed ASSO in the uterine cavity and muscular layer, the whole cervical duct and the right adnexal. She underwent 2 systemic chemotherapy sessions after the surgery. The chemotherapy regimen was ifosfamide 2.5 g day 1 to 3, with liposomal doxorubicin 40 mg day 1.

**Outcomes:**

The final diagnosis was uterine ASSO, International Federation of Gynecology and Obstetrics stage IVa. The patient has been following-up so far, with no progression.

**Lessons:**

Review of the case indicated that history of long-term oral short-acting contraceptives and giant endometrial polyps may be the high-risk factors for UA. For patients with high-risk factors, the follow-up ultrasound scan should be more frequently conducted. Moreover, 3D-ultrasound, MRI and outpatient hysteroscopy are recommended for routine screening. Placement of levonorgestrel-releasing intra-uterine system after hysteroscopy may be an effective intervention for patients with a high risk of giant polyps. Cluster of Differentiation 10, Estrogen receptor, Progesterone receptor, and nuclear antigen may be predictors for prognosis and selection of individualized treatment program.

## 1 Introduction

Uterine adenosarcoma (UA) is a mixed tumor of the uterus consisting of a benign glandular epithelium and a malignant mesenchymal component, which was originally reported as Mullerian adenosarcoma by Clement and Scully in 1974.^[1–2]^ UA is a rare malignancy, which accounts for 8% of all uterine sarcomas and <0.2% of uterine malignant tumors.^[3]^ If the sarcomatous part occupies >25% of the tumor volume, it is referred to as sarcomatous overgrowth. UA is rare in young women and has a good prognosis, but adenosarcoma with sarcomatous overgrowth (ASSO) is more aggressive and associated with worse clinical outcomes. Uterine ASSO is an aggressive disease with a higher recurrence and death rate.^[4–7]^ Currently, there is a lack of specific clinical examination for UA. Its rarity and nonspecificity in clinical presentation, laboratory, and imaging examinations frequently result in misdiagnosis and missed diagnosis. Therefore, it is critical to identify the risk factors related to this disease and conduct effective follow-up and intense screening for high-risk patients, as well as relevant interventions for high-risk factors. In addition, the analysis of relevant prognostic indicators is also important. Herein, a case of a young female with uterine ASSO at stage IVa is reported.

## 2 Case report

**Figure 1:**
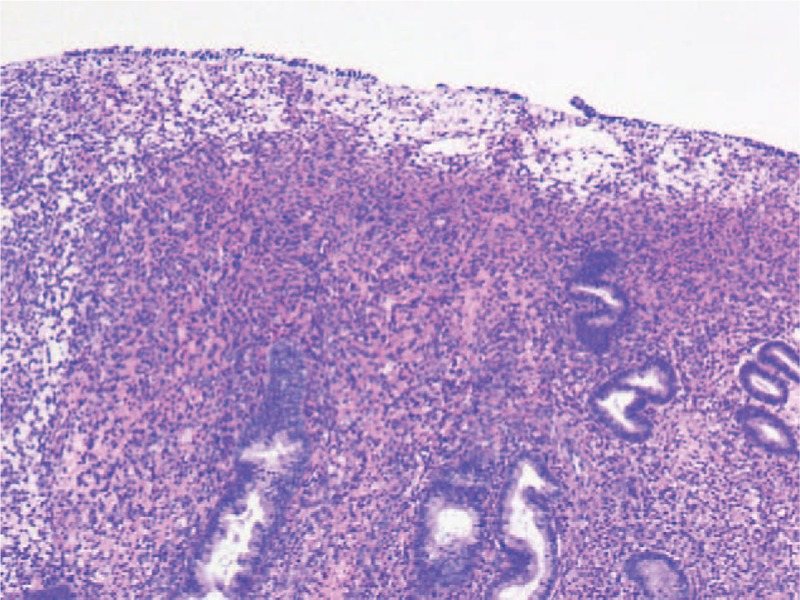
Pathological section showing endometrial polypoid hyperplasia.

**Figure 2:**
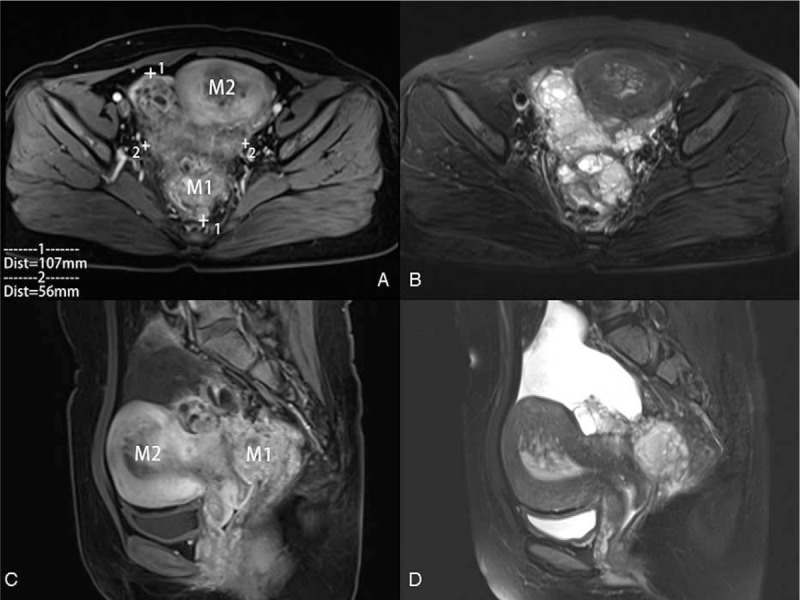
Gadolinium-enhanced T1-weighted images axial (A) and sagittal (C), T2-weighted image axial (B), and sagittal (D) of pelvic MRI showing an irregular mass of the right adnexal (M1) of about 56∗107 mm^2^ with low-signal and partial high-signal and without distinct border with the rectum; an uterine cavity's ill-defined mass with heterogeneous signal (M2), rooting in the myometrium of the posterior wall of the uterus, without distinct border with the uterine wall. MRI = magnetic resonance imaging.

A 29-year-old female, 1-0-0-1, was admitted to our hospital on September 22, 2016, due to extended menstrual period for 6 months. She had a half a year history of regular oral short-acting contraceptive usage and denied a history of other major illnesses. Gynecological examination indicated that the uterus was enlarged to the size of 4 weeks of gestation and several hard nodules could be felt in the posterior wall of the uterus. The ultrasound results indicated that the uterus was enlarged, and a heterogeneous echo of about 71∗18 mm^2^ could be seen from the uterine cavity to the cervical canal. After adequate evaluation and informed consent, endometrial polypectomy under hysteroscopy was performed based on the diagnosis of endometrial polyp and adenomyosis. During the operation, a large ligule vegetation of about 70∗50 mm^2^ was found in the posterior uterine wall near the fundus of the uterus, which was completely resected and sent for pathological examination. The pathological diagnosis of curettage tissues was endometrial polyp (Fig. 1). Regular ultrasound scan postoperation indicated thickened endometrium and an ill-defined mass with continuous enlargement in the myometrium of the posterior wall of the uterus, which was considered as an adenomyoma (Table 1). The patient refused further hysteroscopic exploration, other treatment and follow-up for 1 year since November 2017. On December 27, 2018, the patient was re-admitted due to lower abdominal distension for over 1 week. She has been taking regularly oral short-acting contraceptives for the past 2 years. A gynecological examination revealed that her uterus had enlarged to the size of 8 weeks of gestation. Besides, a mass of approximately 120∗100 mm^2^ was found in the right adnexal area, which extended across and was attached to the uterine body, with unclear boundary and poor mobility. Serum levels of carcinoma antigen 125 was 141.7 U/mL and estradiol was 212.81 pmol/L. Ultrasound scans revealed an enlarged uterus, and a 130∗71∗78 mm^3^ heterogeneous echo cluster was observed in the right annex area and pelvis, which was attached to the right posterior wall of the uterus. The mass was cystic and solid with abundant blood flow in the solid part (RI:0.8). Magnetic resonance imaging (MRI) of the pelvis revealed an irregular mass shadow of about 12∗56∗107 mm^3^ (width × depth × length) with mixed high signal on T2WI, low signal shadow and partial high signal on T1WI in the right annex area, without distinct border with the rectum, moreover, an uneven intrauterine echo that has no obvious boundary with uterine wall (Fig. 2). Lung computed tomography (CT), upper abdominal CT, and gastroenteroscopy showed no abnormality. Right ovarian cancer and adenomyoma were initially considered based on morphology.

**Table 1:**
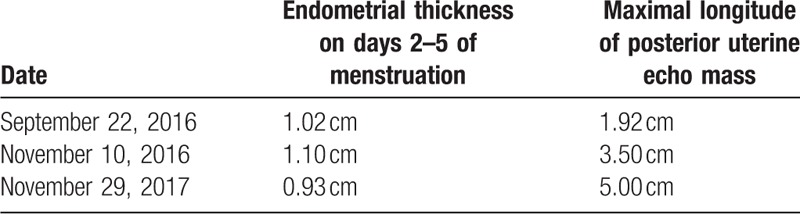
Ultrasound follow-up data.

**Figure 3:**
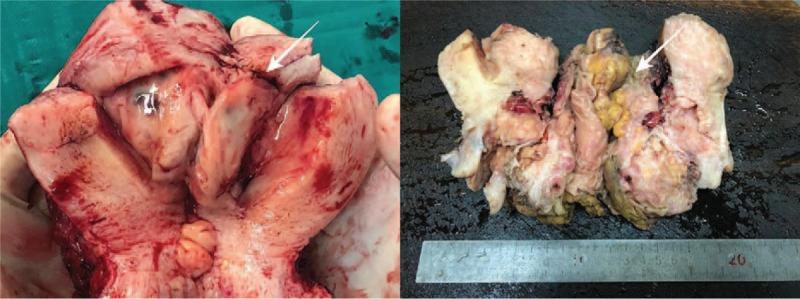
Cut specimen of the uterus showing a soft mass with local bleeding (white arrow).

**Figure 4:**
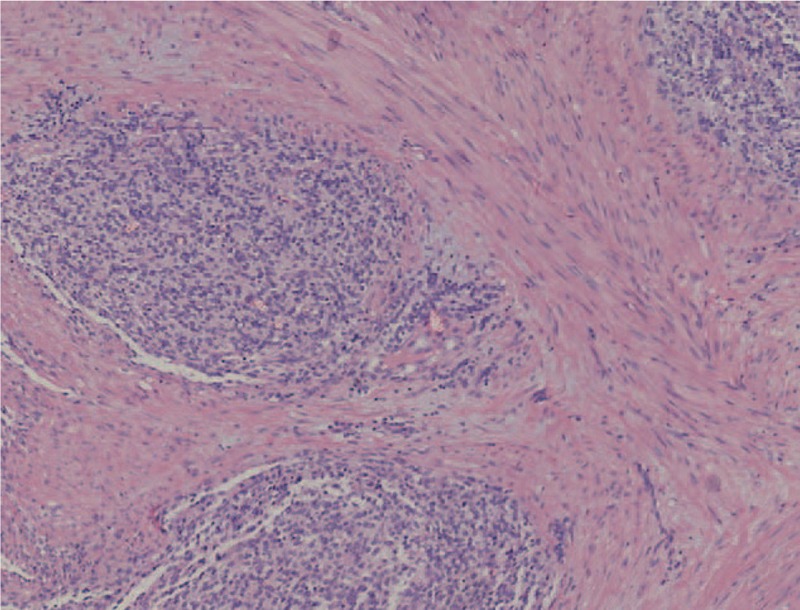
Pathological section showing the tumor was composed of spindle and ovoid cells, which were cuff-shaped around the glands and chondroid matrix could be seen in the interstitium.

Exploratory laparotomy was performed after explaining the patient's condition and receiving consent of the patient and her family, with a clinical preoperative diagnosis of right ovarian cancer and adenomyoma. Laparotomy revealed a mass of approximately 80∗90∗90 mm^3^ in the right ovary, which closely adhered to the posterior lobe of the right broad ligament, the right peritoneum, and the surrounding intestine; the Douglas pouch was completely closed; a mass of 50∗60∗60 mm^3^ in the anterior rectum was densely adhered to the posterior uterine wall and the posterior cervix lip; the uterine enlargement was the size of 50 days gestation and the left ovary was slightly increased with adhesion to the posterior wall of the uterus; scattered implantation lesions of varying sizes were found in the peritoneum, appendix, and omentum. There were no obvious implantation lesions on the surface of the liver, diaphragm, and spleen. In addition, about 300 mL of pale bloody ascites was found in the abdominal cavity (Fig. 3). The patient subsequently received transperitoneal retroperitoneal pelvic combined with total viscera resection, including uterus, bilateral appendages and rectum, omentectomy, appendectomy, lymphadenectomy, and ileostomy. Postoperative pathology examination indicated that the tumor was composed of spindle and ovoid cells, which were cuff-shaped around the glands. Tumor cells showed minor to moderate atypia, and mitotic phase >10/10HPF with hemorrhage in some areas, and chondroid matrix could be seen in the interstitium (Fig. 4). Immunohistochemistry revealed CK7 (epithelium +); interstitial: Vim (+), Cluster of Differentiation 10 (CD10) (+), Smooth muscle actin (−), Desmin (−), Calponin (−), P16 (+), Estrogen receptor (ER) (+ 80%), Progesterone receptor (PR) (+ 90%), Inhibin-alpha (−), Wilms tumor 1 (+), P53 (<3% +), nuclear antigen (Ki-67) (+ 15%); and blood vessels: CD34 (+), CD31 (+). Postoperative pathology confirmed adenosarcoma with sarcomatoid overgrowth of the uterine cavity and muscular layer, the whole cervical duct and the right adnexal. Tumor invaded into the whole rectal wall and the greater omentum, and tumor embolus was found in the vascular system. There was no metastasis in the lymph nodes and no tumor cells in the peritoneal wash. In this case, the tumor cells and mitotic phase as well as the pleomorphism of tumor nuclei were consistent with the diagnostic criteria of uterine ASSO. The final diagnosis was uterine ASSO, International Federation of Gynecology and Obstetrics stage IVa (Table 2). The patient underwent 2 systemic chemotherapy sessions on February 27, 2019, and March 23, 2019, respectively, after the surgery. The chemotherapy regimen was ifosfamide 2.5 g on day 1 to 3, with liposomal doxorubicin 40 mg on day 1. During the interval of chemotherapy, the patient was treated with granulocyte colony-stimulating factor. The patient has been following-up for 4 months by performing abdominal CT and MRI, with no progression. The patient also did not experience any discomfort during the follow-up time.

**Table 2:**
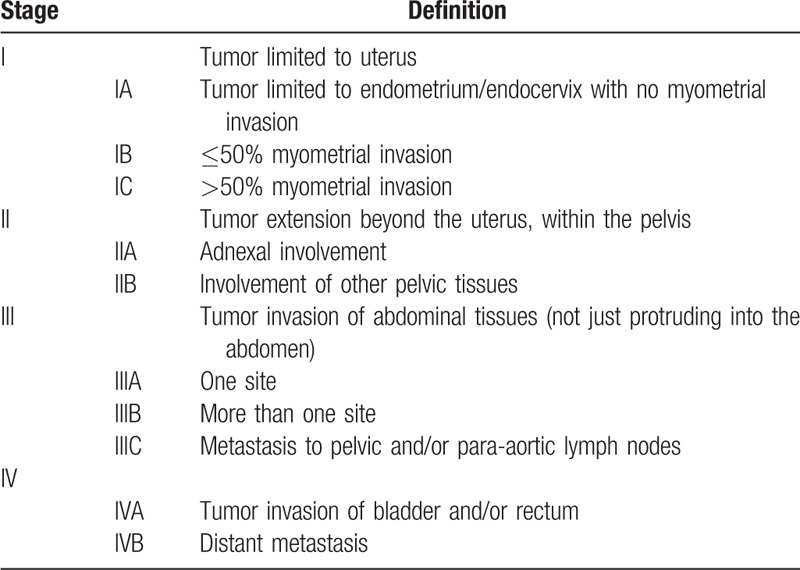
International Federation of Gynecology and Obstetrics staging for uterine adeno-sarcomas, 2009.

## 3 Discussion

Adenosarcoma typically presented as a well-demarcated large polypoid mass occupying the endometrial cavity, and occasionally protruding into the vaginal cavity.^[8]^ It is easily missed and misdiagnosed due to the lack of specific clinical manifestations. Mullerian adenosarcoma was first reported in 1974.^[2]^ According to the current literature, its diagnosis mainly depends on pathological examination due to a lack of auxiliary examination for highly specific abnormality. Uterine ASSO is an aggressive disease with a higher recurrence and death rate. Therefore, actively screening for patients with high-risk and early intervention for high-risk factors are crucial. Certain immunohistochemical may have reference significance in the prediction of prognosis and the selection of individualized treatment schemes.

### 3.1 Risk factors

Prior pelvic irradiation, hyperestrinism, tamoxifen treatment, obesity, and diabetes have been described as risk factors for the development of this biphasic tumor. Also, adenosarcoma may arise from endometriosis and adenomyosis.^[8,9]^ Adenosarcoma of the uterus is suggested to be associated with long-term oral contraceptive use. Tjalma and Michener reported a rare case in 2005 of a 41-year-old patient with UA who was using contraceptive pills for the last 17 years.^[10]^ They considered that the development of adenosarcoma in this patient was related to the long-term use of oral contraceptives. Our patient had a history of regular oral short-acting contraceptive use within 6 months before the first operation and for nearly 2 years after the first operation. The patient's estrogen level was significantly higher than normal. Therefore, the patient's disease may be related to oral short-acting contraceptives. Long-term oral short-acting contraceptive use may be a risk factor for UA, and the underlying mechanism may be the high estrogen level in the body. Further biological and clinical studies are needed to clarify this relationship.

Notably, the size of the polyp (70∗50 mm^2^) was considerably large at the time of the patient's first surgery. Some scholars considered endometrial polyps >1 cm as “large polyps,” while those >4 cm, which are exceedingly rare, as “giant polyps.”^[11]^ Wang et al identified that polyps measuring >1.0 cm were associated with malignancy.^[12]^ Elfayomy and Soliman stated that the polyp volume >10 mL and polyp number were associated with malignancy of endometrial polyps.^[13]^ However, the association between large polyps and UA has been rarely reported. Our patient had a large endometrial polyp in the posterior uterine wall 2 years ago, after which uterine ASSO occurred. The imaging and specimen sections clearly indicated that part of the lesion was located at the posterior uterine wall. So we believed that the patient's previous history of giant polyps may be related to adenosarcoma. Thus, giant endometrial polyps may be a risk factor for UA. This case provides clinical data for the correlation between UA and the history of giant polyps, which needs further clinical verification.

### 3.2 Screening and intervention

In this case, the patient had a history of giant polyps 2 years ago, and only routine ultrasound reexamination was performed after the operation. In addition, no effective screening or other systematic treatment was implemented during the year of missed follow-up, which may be an omission in this treatment process. Therefore, in addition to identifying high-risk factors, it is critical to conduct effective screening and intervention for patients with high-risk factors.

Ultrasound scan is a common imaging examination because of its noninvasiveness and convenience. However, there are many similarities between the ultrasonographic characteristics of UA and other uterine lesions.^[14]^ Ultrasound scan has limited significance in the diagnosis of UA. Most ultrasonographic manifestations of UA in utero are substantive (88%) and cystic and solid (8%), usually with low echo and medium echo, while those located outside the uterus usually show hypoechoic cystic.^[15]^ In this case, ultrasonography indicated large cystic solid space outside the uterus, which was consistent with previous reports. The patient underwent endometrial polypectomy 2 years ago, and postoperative ultrasound examination was regularly conducted for 1 year (Table 1). The previous follow-up data indicated endometrial thickening and progressive increase in the echo group with unclear boundary in posterior wall of the uterus. This suggested that increasing the frequency of ultrasound follow-up in patients with high-risk factors and dynamic observation may be helpful for early detection of abnormal signs. Abou-Salem et al considered that the sensitivity and specificity of 3D-ultrasound in the diagnosis of subendometrial fibroids, endometrial polyps, and endometrial hyperplasia were 92% and 89%, respectively, which were significantly better than that of 2D-ultrasound.^[16]^ Although the role of 3D-ultrasound in gynecological tumor screening has not been widely confirmed, most UAs initially present as a large intrauterine mass. By reviewing the clinical data of the patient, a large intrauterine occupying mass was found in the postoperative specimen section, which was consistent with the manifestation of UA. However, 2D-ultrasonography scan did not indicate obvious intrauterine lesions. We hypothesized that for patients with high-risk factors for UA, it would be useful to use 3D-ultrasonography for timely evaluation.

As compared to ultrasound, MRI is more accurate for preoperative diagnosis and clinical staging. Adenosarcomas of the uterus on MRI are characterized by marked enlargement of the uterus and thinning of the myometrium, with a polypoid mass protruding from the uterine cavity into the cervix. The polypoid mass contains solid components with a high signal intensity. ASSO may present with myometrial invasion and relatively low signal intensity on high b-value diffusion-weighted imaging suggesting that the tumor is low grade.^[17]^ MRI of our patient was consistent with the literature description. With the accumulation of more cases, further summarizing the MRI characteristics of UA will be useful for early diagnosis.

In addition to routine imaging examination, hysteroscopy is also an important examination. Hysteroscopy is invasive and its clinical application is limited. However, the advantage of hysteroscopy lies in examining intrauterine lesions under direct vision and directly conducting biopsy. Hysteroscopy is reported to be a cheap, outpatient 1 step “see and treat” procedure. It causes low pain scores, high patient satisfaction, and high cure rate.^[18]^ Therefore, timely hysteroscopy is necessary for the patients with high-risk factors, especially those with thick endometrium and a history of giant endometrial polyps. In a case report, Li et al considered that post-hysteroscopic progesterone hormone therapy had a favorable clinical effect in treating endometrial polyps as it could effectively prevent the recurrence of endometrial polyps, relieve the level of hemoglobin and reduce endometrial thickness.^[19]^ Therefore, placement of levonorgestrel-releasing intra-uterine system (LNG-IUS) to prevent recurrence post-operation may be a suitable intervention for patients at high risk of UA with giant endometrial polyps.

### 3.3 Prognostic predictors

UA generally has low malignancy with a good prognosis. Stage I adenosarcomas without sarcomatous overgrowth have a good prognosis, with a 5-year overall survival up to 60% to 80%.^[3]^ There is no clear guidance on whether adjuvant radiotherapy and chemotherapy is needed for patients with early-stage, especially for adolescents and women of childbearing age, which is likely to lead to over-treatment or inadequate treatment. Hence, it is particularly important to individualize the prognosis based on corresponding pathophysiological indicators. Clinical stage is the most important factor for prognosis. The 5-year survival rate for patients with stage I adenosarcoma was 79%, decreasing to 48% for patients with stage III disease.^[20]^ For patients with earlier stages, there is a significant correlation between adenosarcoma overgrowth and prognosis. There were differences in 5-year survival rates between patients with or without adenosarcoma overgrowth (50%–60% vs 69%–80%) and in median total survival time (55.4 months vs 112.4 months).^[6]^ The most important prognostic factors of UA are age, sarcomatous overgrowth, myometrial and lymphovascular invasion, and lymph node involvement.^[21]^ The clinical stage of our patient was stage IVa. Sarcomatoid overgrowth and intravascular tumor thrombus were indicated in pathology. Hence, postoperative chemotherapy was performed. Some indicators in immunohistochemistry, such as CD10, ER, PR, and Ki-67, have predictive significance for tumor malignancy and prognosis. CD10 is a common immunohistochemical indicator for adenosarcoma, with a positive expression rate of 70% to 100%. Low or lack of expression of CD10 often occurs (28%) in uterine ASSO. Meanwhile, the expression of ER/PR is related to CD10 and uterine ASSO.^[22,23]^ The positive expression of CD10 in our patient, and the high expression of ER/PR were consistent with previous reports. The patient was pathologically confirmed as uterine ASSO. The absence of distant metastasis may be related to the high expression of CD10 and ER/PR, which have reference significance for the prognosis of UA, but the specificity and sensitivity need to be confirmed by large sample data. Ki-67 is a nuclear antigen that reflects the state of cell proliferation. The higher the positive ratio, the higher is the cell proliferation activity, and the faster is the tumor growth. Ki-67 is often used as an indicator to judge the degree of tumor malignancy in soft tissue tumors. Based on follow-up of cases of UA with FIGO stage I, a study found Ki-67 ≤20% in the better prognosis group and Ki-67 >30% in the worse prognosis group.^[24]^ In this case, postoperative pathology indicated that Ki-67 was 15%, suggesting a relatively good prognosis. However, it has only been 4 months since the operation, and the patient is undergoing adjuvant chemotherapy, with no recurrence. Continued follow-up would be conducted to verify the effectiveness of the corresponding physiological and pathological predictors. Very few cases of uterine ASSO have been reported, so large samples of clinical data are needed to explore the corresponding clinicopathological indicators for predicting the prognosis of the disease, to achieve individualized treatment.

## 4 Conclusion

Uterine ASSO is highly invasive and prone to relapse after surgery. Due to its nonspecific clinical characteristics, it is prone to misdiagnosis and missed diagnosis in the early stage. Long-term oral short-acting contraceptives may be a risk factor for UA and the underlying mechanism may be the high estrogen levels in the body. Polyp volume may be related to the occurrence of UA. For patients with giant polyps, placement of LNG-IUS after hysteroscopy may reduce the possibility of disease recurrence and malignant transformation. For patients with high-risk factors, the follow-up ultrasound should be more frequently conducted. Three-dimensional ultrasound examination, MRI, and outpatient hysteroscopy may be more effective in screening. Besides, with the accumulation of cases, immunohistochemical indexes such as CD10, ER, PR, and Ki-67 may have reference significance in the prediction of prognosis and the selection of individualized treatment schemes. Case reports in the literature are limited because of the low incidence of UA, especially of uterine ASSO. At present, it is difficult to establish high-level confirmation of uterine ASSO, and its biological behavior and clinical prognosis need to be further analyzed after accumulating more cases.

## Author contributions

**Data curation:** Xin-He Shi.

**Formal analysis:** Hua-Di Yang.

**Investigation:** Hua-Di Yang.

**Software:** Hua-Di Yang.

**Writing – original draft:** Bin Wang, Hui Li.

**Writing – review and editing:** Hui Li.
